# Hydrogen Bonding in Amorphous Indomethacin

**DOI:** 10.3390/pharmaceutics16081002

**Published:** 2024-07-29

**Authors:** C. J. Benmore, J. L. Yarger, S. K. Davidowski, C. D. Shrader, P. A. Smith, S. R. Byrn

**Affiliations:** 1 X-ray Science Division, Advanced Photon Source, Argonne National Laboratory, Argonne, IL 60439, USA; 2 School of Molecular Sciences, Arizona State University, Tempe, AZ 85281, USA; jeff.yarger@asu.edu (J.L.Y.); steve.davidowski@vextscience.com (S.K.D.); shradec@purdue.edu (C.D.S.); 3 Improved Pharma, West Lafayette, IN 47906, USA; pam.smith@improvedpharma.com (P.A.S.); sbyrn@purdue.edu (S.R.B.); 4 Department of Industrial and Physical Pharmacy, Purdue University, West Lafayette, IN 47906, USA

**Keywords:** amorphous, pair distribution function, indomethacin, X-ray diffraction, Monte Carlo simulation

## Abstract

Amorphous Indomethacin has enhanced bioavailability over its crystalline forms, yet amorphous forms can still possess a wide variety of structures. Here, Empirical Potential Structure Refinement (EPSR) has been used to provide accurate molecular models on the structure of five different amorphous Indomethacin samples, that are consistent with their high-energy X-ray diffraction patterns. It is found that the majority of molecules in amorphous Indomethacin are non-bonded or bonded to one neighboring molecule via a single hydrogen bond, in contrast to the doubly bonded dimers found in the crystalline state. The EPSR models further indicate a substantial variation in hydrogen bonding between different amorphous forms, leading to a diversity of chain structures not found in any known crystal structures. The majority of hydrogen bonds are associated with the carboxylic acid group, although a significant number of amide hydrogen bonding interactions are also found in the models. Evidence of some dipole–dipole interactions are also observed in the more structurally ordered models. The results are consistent with a distribution of Z-isomer intramolecular type conformations in the more disordered structures, that distort when stronger intermolecular hydrogen bonding occurs. The findings are supported by ^1^H and ^2^H NMR studies of the hydrogen bond dynamics in amorphous Indomethacin.

## 1. Introduction

Active pharmaceutical ingredients can exist in a variety of solid forms with a range of different intermolecular interactions that affects both their bioavailability and structure [1,2,3]. Here, we consider the hydrogen bonded structure and properties of amorphous Indomethacin, which has been studied extensively in the literature due to the increased solubility over its crystalline forms [4,5,6]. Several previous studies have compared the effects of different amorphization methods and storage conditions on the properties and stability of Indomethacin. Cowley and Zografi [7] cryogenically ground five different starting crystal phases to produce amorphous forms that exhibited significant differences in stability. Yoshika et al. [8] showed that the crystallization rates and mechanisms differ above and below the glass transition temperature. Andronis et al. [4,9] studied the effects of water which changed the surface properties, crystallization rates, and polymorph formation. Greco et al. [10] investigated the effects of processing and annealing on the dissolution of amorphous Indomethacin. Karmwar et al. [11] prepared amorphous samples by melt quenching, spray drying, ball milling, and cryo-milling that yielded different shapes in their X-ray halos indicating a variation in packing between molecules.

The Indomethacin molecule (C_19_H_16_ClNO_4_) comprises a largely hydrophobic indole and chlorobenzyl groups and several hydrophilic groups: namely an amide, methoxyl, and a carboxylic acid [12]. γ-Indomethacin is the stable crystalline form which exists only with Z isomers [13], where hydrogen bonded dimers are connected through their carboxylic acid groups. α-Indomethacin is a denser metastable crystalline form comprising three different isomers Z, E, and α3 [14], and δ-Indomethacin has recently been found to consist of a dimer of the Z and E isomers [15]. Three new polymorphs obtained from aqueous suspensions have also yet to be characterized [15,16]. Our previous X-ray study on amorphous Indomethacin found a range of disordered structures denoted I through V [17]. In the amorphous forms, the chlorobenzyl ring showed evidence of distinct isomer orientations in samples 1 and II, where the first sharp diffraction peak (FSDP) and medium range ordering was found to be lower. However, for those amorphous samples with no preferred torsion angles of the chlorobenzyl ring (samples IV and V), enhanced medium range order attributed to intermolecular hydrogen bonding was observed, and this was reflected as a 20% increase in the intensity of the FSDP.

**Figure 1:**
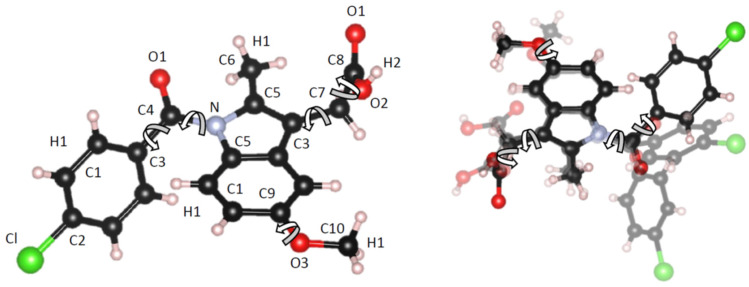
(**Left**) Starting conformation of the Indomethacin molecule used in our EPSR models together with labels of the different atom types. (**Right**) Overlay of Z, E, and α3 isomers to illustrate the choice of the five specified allowed rotations denoted by curved arrows. Carbon atoms are shown as black, nitrogen as blue, oxygen as red, chlorine as green, and hydrogen as white.

The isomers of Indomethacin can be primarily identified from the hindered rotation of the partial double bond between the N1 and C2 atoms (see Figure 1). This can lead to much more potent anti-inflammatory activity associated with the Z-isomer compared to the E isomer [12]. Therefore, in this study, we have performed Empirical Potential Structure Refinement modeling of our previously reported high-energy X-ray diffraction data on different amorphous Indomethacin samples, to investigate the relation between molecular conformation and the range of intermolecular hydrogen bonding interactions. Previous EPSR studies have demonstrated subtle but important hydrogen bonding differences between liquid and amorphous pharmaceuticals and excipients [18,19]. In addition to the solid state NMR (ssNMR) studies on samples I–V that have previously been reported [17], new experiments with partially deuterium exchanged amorphous indomethacin are reported to further investigate the possible molecular conformations and intermolecular bonding configurations.

## 2. Materials and Methods

Crystalline γ-Indomethacin (>98% from Tokyo Chemical Industry) was used as received without further purification. The acetonitrile (99.9% HPLC grade, Concord Technology (Tianjin, China) and the deuterium oxide (99.9% atom D, Cambridge Isotopes Laboratories) were used as received without further purification. To prepare indomethacin deuterated at the exchangeable acid position, 300 mg of indomethacin (0.8 mmol) was dissolved in 7 mL of a 70:30 mixture of acetonitrile and deuterium oxide (110 mmol) under mild heating and stirring. The solution was allowed to stir on a hot plate under mild heating for approximately 3 h prior to solvent removal by dry nitrogen purge, followed by vacuum drying, resulting in the α crystal form. To obtain the γ form, the indomethacin was recrystallized by dissolving in the minimum amount of warm acetonitrile and allowing the solution to slowly cool to room temperature, followed by storage overnight at 5 °C. The amorphous indomethacin samples were prepared by melt quenching with liquid nitrogen.

The ssNMR spectra were collected using a 400 MHz Varian VNMRS system equipped with a 1.6 mm triple resonance HXY probe configured for ^1^H-^13^C-^2^H operation with resonant frequencies of 399.739, 100.524, and 61.363 MHz, respectively. The ^1^H ssNMR spectra were collected with a 1.75 μs ^1^H 90-degree pulse, a 30 s recycle delay, and a magic angle spinning (MAS) speed of 20 kHz. The ^2^H spectra were collected with a 1.75 μs ^2^H 90-degree pulse, a recycle delay of 3 s, 20k scans, an MAS speed of 5 kHz, a sweep width of 500 kHz, and an acquisition time of 8.192 ms. The ^1^H → ^13^C cross polarization (CP)-MAS spectra were collected using a 2.25 μs ^1^H 90-degree pulse, between 1k and 8k scans, a recycle delay of 10 s, an MAS speed of 20 kHz, and a CP contact time of 2 ms. The CP was achieved using a 100 kHz ^13^C spin-lock pulse and a ramped power (5%) ^1^H spin-lock pulse optimized to the −1 spinning side band of the Hartmann–Hahn condition (80 kHz). During ^13^C and ^2^H data collection at high power (140 kHz), two-pulse phase-modulated (TPPM) ^1^H decoupling with a 3.3 μs pulse width and 8-degree phase offset was used, to improve spectral resolution. The chemical shifts for ^1^H and ^13^C were indirectly referenced to TMS in the solid state by setting the resonances for ^1^H and ^13^C of adamantane to 1.8 ppm and 38.48 ppm, respectively. The ^2^H chemical shifts were referenced by setting the ^2^H resonance of liquid D_2_O to 4.8 ppm. The ^1^H and ^13^C NMR data were processed using VnmrJ 4.2, and the ^2^H NMR data were processed using Topspin 4.1. The ^2^H NMR line shapes were fit and analyzed using DMFit [20].

To investigate the variation in hydrogen bonding between the different amorphous forms of Indomethacin measured in our high-energy X-ray diffraction experiments on beamline 6-ID-D at the Advanced Photon Source, Empirical Potential Structural Refinement (EPSR) modeling [21] was used. The five samples were all prepared by melt quenching and their preparation and characterization has previously been reported in detail by Benmore et al. [17]. It is important to re-iterate here that an accurate data reduction procedure is essential in obtaining the X-ray structure factor S(Q) and associated pair distribution function G(r). A review of the current software available for this purpose has recently been carried out by Gallington et al. [22]. EPSR is a Monte Carlo semi-rigid body type simulation, whereby all atoms on the molecules are defined using harmonic force constants, and angular and dihedral angles are used to describe the molecular geometry and allowed intramolecular rotations [23,24]. The algorithm initially uses Lennard-Jones reference potentials with Coulombic terms to describe the intermolecular interactions. As the simulation progresses, an empirical potential is employed to modify these intermolecular interactions. This term in the potential is determined by taking the difference between the experimental diffraction data and that predicted by the Monte Carlo model. The continuously perturbed potential drives the model structure towards the experimental data by random changes in atomic (molecular) coordinates, including molecular rotations if flexibility of that part of the molecule is allowed, with each step resulting in new configurations. The change is accepted if the potential energy decreases, or with a Boltzmann probability if it is greater, to avoid becoming stuck in local minima. Once good fits are found between the model structures and the measured X-ray structure factor, the simulation is collected over a large number of configurations. When applied to X-ray diffraction data from amorphous pharmaceuticals, the scattering is mainly dominated by the carbon and oxygen atoms that define the molecular geometry and intermolecular pair correlations.

Here, EPSR simulations were performed on 64 molecules within a cubic box under periodic boundary conditions. The starting configuration of our model was constructed from a random array of Z isomer molecules since this is the most common isomer, particularly at low density. In addition, molecular dynamics simulations of amorphous Indomethacin predict that the Z isomer is more favorable than the E isomer by a factor of about 5.7 [1]. To allow for other conformations in the model and improve the fit to the X-ray data, rotations of five molecular groups were enabled, including the rotation of the chlorobenzyl ring. The atom labels for the different atom types used in the simulation and the allowed molecular rotations are illustrated in Figure 1. To prevent unrealistic hydrogen bonding, an additional “soft” minimum distance constraint of 2.6 Å was applied for the oxygen–oxygen (O-O) interactions between adjacent molecules. This constraint increases the repulsive part of the inter-atomic potential but may come to equilibrium at a lower atom–atom distance if necessary, in order to maintain an adequate fit to the data.

The parameters associated with the starting Lennard-Jones reference potentials are shown in Table 1. For simplicity, only the most influential partial charges were employed based on the molecular dynamics simulations of Xiang and Anderson [12], namely, the negative acceptor oxygen and positive carbon charges, with the charge balance placed on the OH donor hydrogen. Best fits to the more disordered high-energy X-ray diffraction signals (from samples I and II) were obtained using a ~5% lower density and the semi-rigid molecular models, whereby rotation about the 5 specified axes was allowed. The more ordered signals from samples IV and V were better fit using rigid Z-isomer molecules with rotations restricted to only a few degrees. Sample III was fit with an intermediate density and all but 5 rotations enabled.

Following initial Monte Carlo equilibration, the empirical potential term was refined to improve agreement with scattering data. Once the goodness-of-fit parameter was minimized between the model and the experimental S(Q), structural data were collected over ensembles of at least 10,000 configurations. While the EPSR fit to the data does not necessarily give a unique structural 3D configuration of molecules, it does provide an important insight into the types of interactions that are likely in the disordered state. Since X-rays are scattered by electrons, the S(Q)s and corresponding PDFs are most sensitive to the heavier atoms and in particular the orientations of the carbon rings, oxygens, and the chlorine atom interactions.

## 3. Results

### 3.1. EPSR Models

**Figure 2:**
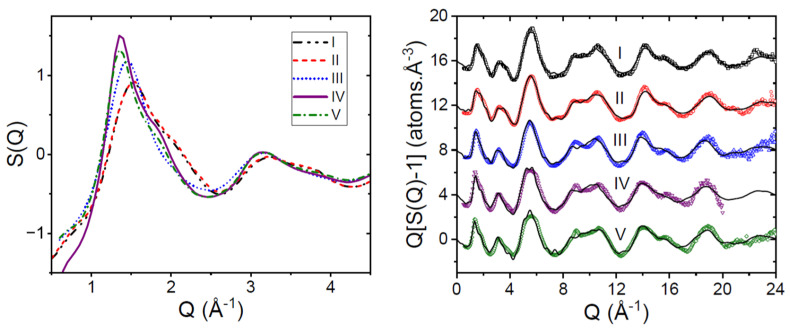
The X-ray structure factors of the five amorphous Indomethacin samples reported by Benmore et al. [17]. (**Left panel**) shows the S(Q) low-Q region encompassing the first sharp diffraction peak. The (**right panel**) shows the entire measured Q-range (circles) using the formalism Q[S(Q)-1] to emphasize high-Q, together with the EPSR model fits from this study (lines).

In order to best fit the periodicity in the high-Q region of the X-ray diffraction pattern, the average intramolecular bond lengths of the molecule needed to be lengthened by 1 to 3% compared to the Z-isomer molecule in the γ-form [14]. This average C-X (where C=C, N or O) bond length is defined in real space by the first peak in the pair distribution function D(r). The results of the fits are shown in Figure 2 and listed in Table 2.

**Figure 3:**
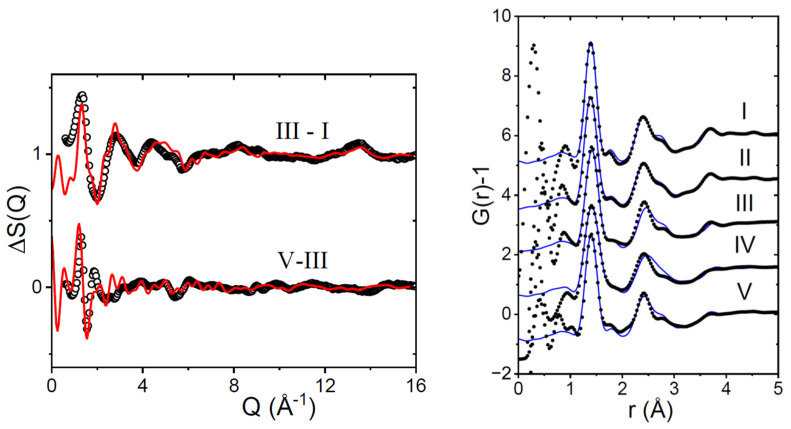
(**Left**) The difference between measured structure factors of amorphous Indomethacin samples (open circles) compared to the same difference between EPSR models (red lines). (**Right**) EPSR fits (blue lines) to the experimental real space X-ray pair distribution function D(r) (black circles) using the different sized Indomethacin molecules as described in Table 2.

The X-ray patterns for the different amorphous samples are qualitatively similar; however, important quantitative differences exist. A strong test of the validity of the different models is demonstrated by taking differences between the less ordered forms (I and II) and more ordered forms (IV and V), as shown in Figure 3. The main difference between the measured amorphous structure factors occurs in the low-Q region, which can be associated with the packing (and hydrogen bonding interactions) between molecules [25].

**Figure 4:**
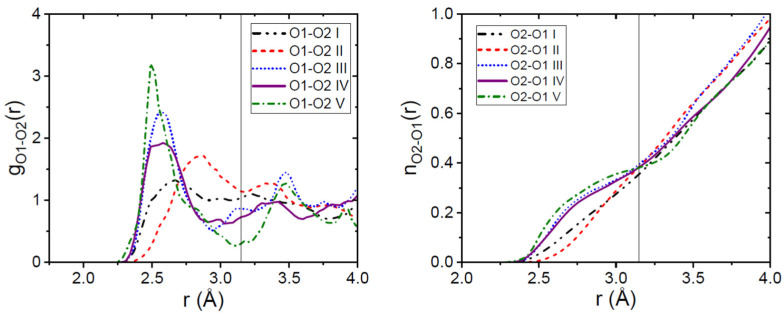
(**Left panel**) The intermolecular carboxyl acid oxygen donor O2-O1 oxygen acceptor partial pair distribution functions from our EPSR models. The (**right panel**) shows corresponding running coordination numbers for the amorphous Indomethacin samples.

The O2H2-O1 hydrogen bonded distance in the γ-form is 2.67Å and is associated with the formation of carboxylic acid dimers. In α-Indomethacin, the structure comprises three molecules in the asymmetric unit, with each molecule having a different conformation, forming a trimer. The trimer comprises a hydrogen-bonded carboxylic acid dimer, with the third molecule forming a hydrogen bond between the carboxylic acid and an amide carbonyl in the dimer, spanning O2H2-O1 distances between 2.59 and 2.74 Å. In contrast, our EPSR models of the amorphous forms show a wide range of O2H2-O1 distances, from 2.50 to 2.85 Å. The partial pair distribution function g_O2O1_(r) in Figure 4 shows the distribution of O2H2-O1 hydrogen bonding interactions as a function of distance in the different amorphous Indomethacin samples. 

The EPSR models for the more-ordered samples (IV and V) exhibit strong hydrogen bonds in the form of a single intense peak at ~2.5 Å, a minimum at 3.2 Å, and second shell maximum at ~3.5 Å. The less-ordered samples (I and II) span a broad (and continuous) range of O2H2-O1 distances from 2.5 to 3.5 Å. This variation in the average number of hydrogen-bonded molecules as a function of distance between different amorphous forms is reflected in the running coordination number, n_O2-O1_(r) in Figure 4. Nevertheless, a value of n_O2-O1_(r)~0.38 was found for all the amorphous samples at a distance of r = 3.2 Å. This compares to the value of 1.0 in the crystalline γ- and α-forms.

**Figure 5:**
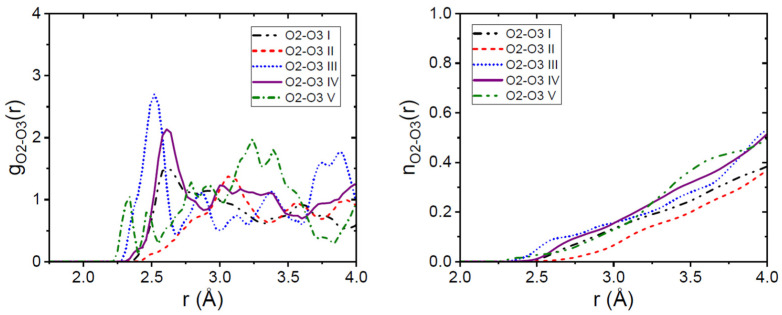
(**Left panel**). The intermolecular carboxyl acid oxygen donor O2-O3 amide oxygen acceptor partial pair distribution functions from our EPSR models. The (**right panel**) shows the corresponding running coordination numbers for the amorphous Indomethacin samples.

In our amorphous models, hydrogen bonding intermolecular interactions were also found between the carboxyl acid oxygen O2 and the O3 amide oxygen, which is not found in crystalline forms and likely arises from the highly disordered arrangement of molecules. Interestingly, the strongest O2-O3 interactions are found in sample III which exhibited the sharpest FSDP, see Figure 5. In other words, it is the sample with the most intermolecular hydrogen bonding of the models with the most flexibility, by allowing all five intramolecular rotations. Although, we note that the most-ordered models, namely III, IV, and V, all exhibit a O2-O3 coordination number, n_O2-O3_(r)~0.2, at a distance of r = 3.2 Å.

In addition, since the amide carbonyl atoms C4 and O1 have large and opposite charges of +0.7 and −0.6 (see Table 1) they have the potential to induce dipole–dipole bonds between adjacent Indomethacin molecules. Indeed, in models IV and V, evidence of short intermolecular C4···O1 distances of ~3.2 Å are found, similar to those observed in the γ-crystalline form, but these interactions are largely absent in models I, II, and III. 

### 3.2. ssNMR Experiments

**Figure 6:**
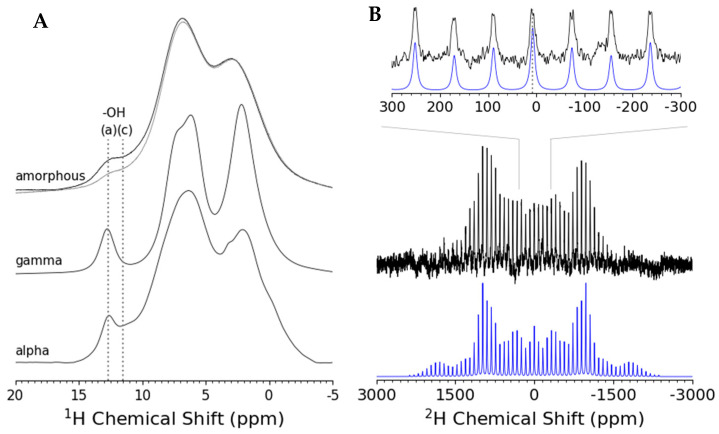
(**A**) ^1^H solid-state MAS (ν_r_ = 20 kHz) NMR spectra of alpha, gamma, and amorphous indomethacin. A selectively acid deuterium-enriched amorphous indomethacin sample was made through ^1^H to ^2^H exchange prior to melt quenching. The ^1^H solid-state MAS (ν_r_ = 20 kHz) NMR spectrum of the (grey) partially acid deuterated amorphous indomethacin sample (d_1_-indomethacin) is overlaid with the (black) standard amorphous indomethacin and shows a reduction in ^1^H signal due to the partial acid deuteration. Dotted vertical lines are shown at 12.7 and 11.5 ppm and labeled (a) and (c), respectively. These are the approximate ^1^H chemical shifts for the acid (COOH) protons in a (a) hydrogen-bonded dicarboxylic acid environment (gamma-indomethacin) and (c) in a weaker hydrogen-bonded carbonyl—carboxylic acid environment. (**B**) ^2^H solid-state MAS (ν_r_ = 5 kHz) NMR spectra (black) and DMFit simulation (Blue) of amorphous d_1_-indomethacin (~50% ^2^H exchanged at the acid site). The best fit ^2^H quadrupolar tensor gave a coupling constant (C_Q_) of 178 kHz with no asymmetry (η = 0) with a ^2^H chemical shift of 8 ppm and a linewidth of 10 ppm. Above the full 2H spectrum and simulation is a zoomed in plot to more clearly show the center band and the first few satellite transitions (black) and associated simulation fit (blue) of amorphous d_1_-indomethacin.

The ^1^H magic angle spinning (MAS) spectra of the natural abundance amorphous and crystalline γ- and α-Indomethacin samples are shown in Figure 6A along with partially deuterium exchanged amorphous indomethacin (overlaid grey spectrum). The reduction in the ^1^H MAS spectral intensity in the 10–14 ppm range for the amorphous d_1_-indomethacin in comparison to the natural abundance amorphous indomethacin is confirmation of the selective acid exchanged deuteration of the sample. The ^1^H MAS spectra clearly indicate that the amorphous indomethacin has hydrogen bonding components that are similar to the dimer carboxylic acid configuration found in γ-Indomethacin (~13 ppm) as well as the carboxylic acid hydrogen bonding to the carbonyl as observed in α-Indomethacin (~11 ppm) [26]. The ^1^H acid region in amorphous indomethacin is broad compared with the crystalline samples due to the larger dispersion of hydrogen bonding and acid environments found in disordered molecular solids (Supplementary Material Figure S1).

The ^2^H MAS NMR of d_1_-Indomethacin (~50% deuterated at the carboxylic acid based on ^1^H MAS NMR integration) allowed for elucidation of dynamic processes and further structural probing of the amorphous indomethacin hydrogen bonding environment (Supplementary Material Figure S2). The ^2^H MAS NMR of amorphous d_1_-Indomethacin is shown in Figure 6B along with a first-order quadrupolar simulation using DMFit to estimate the ^2^H quadrupolar coupling constant [20]. Both the isotropic chemical shift and the quadrupolar coupling constant (C_Q_) can be of interest in studies of hydrogen bonds and any associated exchange or molecular motion dynamics. The deuterium quadrupolar coupling constants for carboxylic acids are typically 140–200 kHz and the quadrupole coupling constant for heavy water and common hydrates is ~220 kHz [27,28]. The residual quadrupolar interaction is directly influenced by translational diffusion, molecular rotation, and exchange dynamics. The observation of a quadrupole coupling constant of 178 kHz for d_1_-Indomethacin indicates that that amorphous indomethacin below T_g_ (at room temperature) does not have any appreciable exchange dynamics and exists as distributions of rigid intermolecular hydrogen-bonded molecular units. The ssNMR measurements were supported by Fourier Transform-Infra Red (FT-IR) spectra of the Indomethacin polymorphs (Supplementary Material Figure S1) and deuterated versus natural abundance Indomethacin (Figures S4 and S5). The carbonyl stretching region contains assignments of characteristic stretching bands for each polymorph and were made in reference to the work of Van Duong, et al. [29].

## 4. Discussion

**Figure 7:**
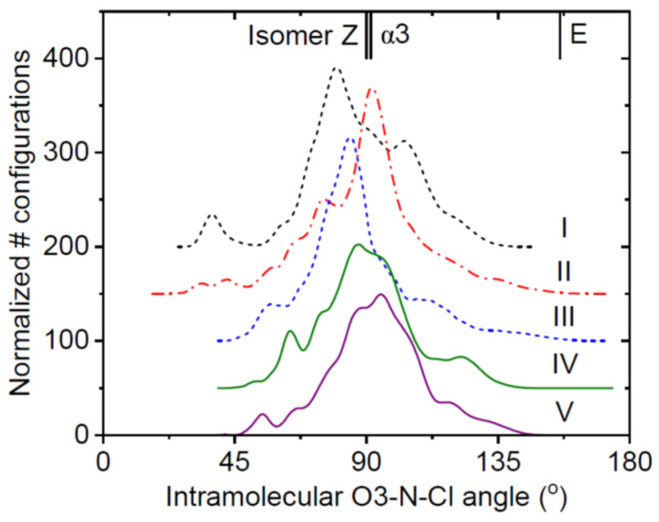
The intramolecular ∠O3-N-Cl angle determined from the EPSR models of the five samples. The angles associated with the three different isomers found in the crystal are denoted on the top axis.

Previous structural characterization methods of crystalline, liquid, and amorphous forms of Indomethacin have included Raman and infra-red spectroscopy [30], nuclear magnetic resonance [28], X-ray crystallography [14], and molecular dynamics simulations [31]. The extraction of the pair distribution function from diffraction measurements provides yet another powerful tool capable of probing both intra- and intermolecular configurations of molecules, especially with regard to the most disordered forms [32,33,34]. The five amorphous Indomethacin samples modeled here have previously been characterized in detail using high-energy X-ray diffraction, nuclear magnetic resonance, Raman scattering, and differential scanning calorimetry [17]. From our EPSR models, we can interrogate the variation in the model structures more thoroughly. A comparison of the ∠O3-N-Cl intramolecular angle of the three different isomers found in α- and γ-Indomethacin are shown in Figure 7, along with the distribution of angles found in the EPSR models for all five amorphous samples. The most structured intermolecular amorphous forms have the broadest range of intramolecular conformations (around ~90°). This is a consequence of our EPSR constraints, since our models of samples IV and V used Z-isomer molecules with rotations limited to only a few degrees. In contrast, our models for our more disordered samples, I, II, and III, allowed for five rotations within the Z-isomer molecules. This additional flexibility resulted in the exhibition of sharper peaks in the ∠O3-N-Cl angle distribution and is attributed to preferred intramolecular conformations. None of the models I, II, or III found configurations with any significant population of E-isomers, suggesting that the pure amorphous forms likely act as better anti-inflammatory agents compared to the α-form. However, it has been pointed out that the distribution of Indomethacin conformations is sensitive to the physical environment, with the Z isomer conformation being preferred in solution and the E-isomer favored in inclusion complexes with β-cyclodextrin [35].

**Figure 8:**
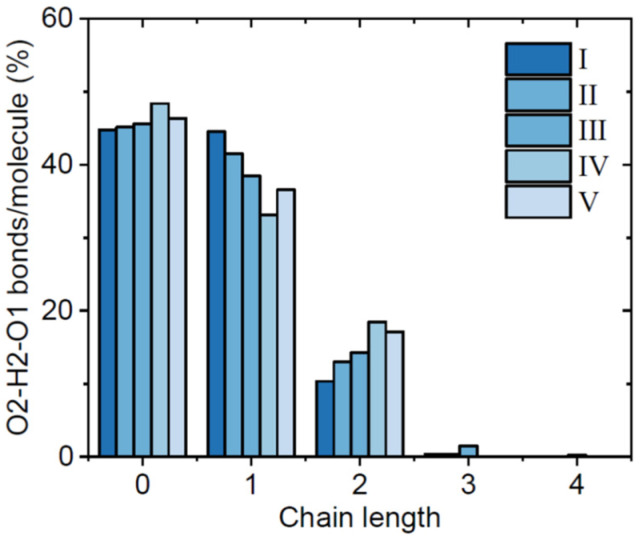
The percent of the number of hydrogen bonds per molecule as a function of chain size for our amorphous Indomethacin samples.

**Figure 9:**
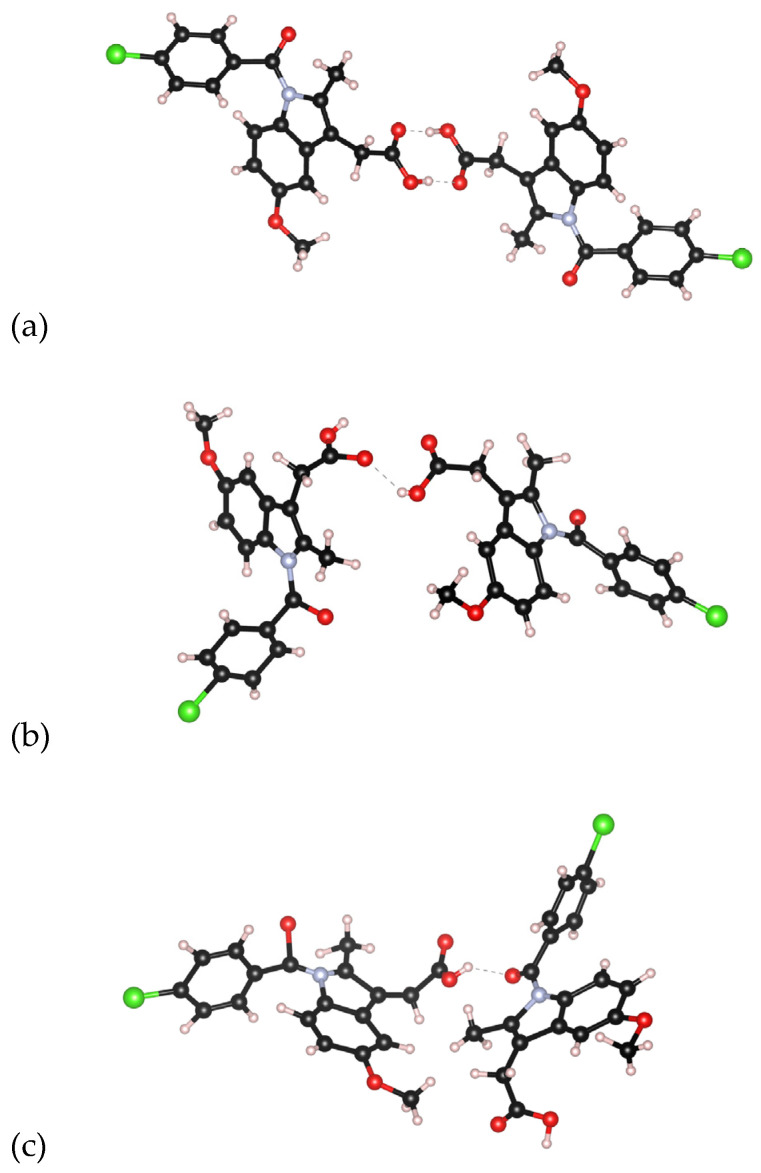
Snapshots of (**a**) a carboxylic acid dimer found in γ-Indomethacin, (**b**) a singly hydrogen-bonded molecular pair, and a (**c**) hydrogen bond between the carboxylic acid and an amide carbonyl found in our EPSR models. Carbon atoms are shown as black, nitrogen as blue, oxygen as red, chlorine as green, and hydrogen as white.

The broad range of molecular conformations observed in our models leads to a high degree of intermolecular structural disorder associated with amorphous solids, and more complex hydrogen bonding patterns compared to the crystalline forms. Previous MD simulations on amorphous Indomethacin [12] have shown a much lower probability of carboxylic dimers than that found in the crystals, and the most readily identified hydrogen bonded patterns were found to be short chains of Indomethacin molecules connected via carboxylic acid bonds. An analysis of the chain size distributions shows a high degree of consistency across all of our EPSR models (see Figure 8), with ~46% of Indomethacin molecules being non-bonded (isolated), and only ~39% bonded to one neighboring molecule via a single hydrogen bond, see Figure 9. The remaining 15% are associated with trimers and bifurcated hydrogen bonds leading to a diversity of chain structures in the amorphous forms. This result compares to 21% non-bonded molecules, 31% singly hydrogen-bonded molecules, and 48% with two or three hydrogen-bonded neighbors in a previously reported molecular dynamics model of 10 mole.% water containing Indomethacin glass [12].

^1^H and ^2^H NMR support the molecular model of amorphous indomethacin having a broad range of molecular conformations and intermolecular structural disorder with complex hydrogen bonding patterns. Furthermore, deuteration and ^2^H NMR are shown as a promising direction for probing hydrogen bonding and molecular structure, motion, and exchange dynamics in pharmaceutical compounds [36]. This is especially relevant with the increased interest in deuterated pharmaceutics [37]. Our future directions involve more closely integrating molecular computations of quadrupolar and chemical shift NMR quantities from ab initio computational model molecular configurations to better combine the experimental and computational elucidation of complex distributions of conformations and intermolecular distributions common to amorphous pharmaceutical compounds.

## 5. Conclusions

Semi-rigid molecular models fitted to five amorphous Indomethacin X-ray diffraction patterns have been interrogated to determine the main similarities and differences between the amorphous samples. All intramolecular conformations are consistent with a wide distribution of configurations similar to the Z-isomer but not the E-isomer. The majority of amorphous Indomethacin hydrogen bonds were found to include the donor carboxylic acid group and one of several hydrogen bond acceptor oxygen sites including the amide carbonyl oxygen, the methoxyl, and the carboxylic acid. Consequently, a wide range of hydrogen bonding strengths and interactions are found across the different models resulting in complex interaction patterns, although the primary hydrogen bond is found to be via carboxylic acid donor O2-O1 acceptor interactions. To a lesser extent, O2-O3 amide hydrogen bonding interactions were also observed, and C4-O1 dipole–dipole interactions occurred in the more structurally ordered models. Overall, the majority of Indomethacin molecules were found to be either isolated (~46%) or form singly hydrogen-bonded dimers (39%). Our amorphous models were consistent with our previous findings that there is competition between preferred intramolecular conformations and stronger intermolecular hydrogen bonding. From a wider perspective, this study shows that the method of EPSR modeling of high-energy X-ray diffraction patterns from amorphous pharmaceuticals is a powerful tool in exploring the range of possible molecular structures.

## Figures and Tables

**Table 1:** Starting Lennard-Jones parameters and partial charges.

Atom	ε (kJ/mol)	σ (Å)	Partial Charge, Q
O1 (acceptor), O2 (donor)	0.65	3.1	−0.6
O3 (acceptor)	0.65	3.1	−0.4
C4, C8	0.8	3.7	+0.7
H2	0	0	+0.8
H1	0	0	0.0
C1, C2, C3, C5, C6, C7, C9, C10	0.8	3.7	0.0
Cl	0.8	3.2	0.0

**Table 2:** EPSR simulation box parameters, densities, and average C-C, C-O, and C-N intramolecular bond lengths compared to the crystal used in this study.

Sample	Atomic Number Density (AtomsÅ^−3^)	Number of Rotations within Molecule	Intramolecular C-X Bond Length or Expansion (Å)
γ-form (crystal)	0.0952	None (Z isomer fixed geometry)	1.380 (initial bond)
I	0.0900	5	+0.015
II	0.0900	5	+0.015
III	0.0925	5	+0.036
IV	0.0950	None (slight variation)	+0.041
V	0.0950	None (slight variation)	+0.036

## Data Availability

The raw data supporting the conclusions of this article will be made available by the authors on request.

## Supplementary Materials

The following supporting information can be downloaded at https://www.mdpi.com/article/10.3390/pharmaceutics16081002/s1, Figures S1 and S2: ^1^H-^13^C CP-MAS ssNMR spectra. Figure S3: FT-IR spectra of Indomethacin polymorphs. Figures S4 and S5. FT-IR spectra of deuterated vs. natural abundance γ-Indomethacin.
