# Supplementary material for: Hydrogen Bonding in Amorphous Indomethacin
Source: Pharmaceutics. 2024 Jul 29;16(8):1002. doi: 10.3390/pharmaceutics16081002 (PMC11359274; doi:10.3390/pharmaceutics16081002)
Supplement: Supplementary file 1 [file pharmaceutics-16-01002-s001.zip › pharmaceutics-3112437-supplementary.pdf]

Supplementary Material

Hydrogen Bonding in Amorphous Indomethacin

C. J. Benmore [1,2,*], J. L. Yarger [2], S. K. Davidowski [2], C. D. Shrader [2], P. A. Smith [3] and S. R. Byrn [3,4]

[1] X-ray Science Division, Advanced Photon Source, Argonne National Laboratory, Argonne, IL 60439, USA

[2] School of Molecular Sciences, Arizona State University, Tempe, AZ 85281, USA; jeff.yarger@asu.edu (J.L.Y.); steve.davidowski@vextscience.com (S.K.D.); shradec@purdue.edu (C.D.S.)

[3] Improved Pharma, West Lafayette, IN 47906, USA; pam.smith@improvedpharma.com (P.A.S.); sbyrn@purdue.edu (S.R.B.)

[4] Department of Industrial and Physical Pharmacy, Purdue University, West Lafayette, IN 47906, USA

* Correspondence: benmore@anl.gov; Tel.: +1-630-2524207

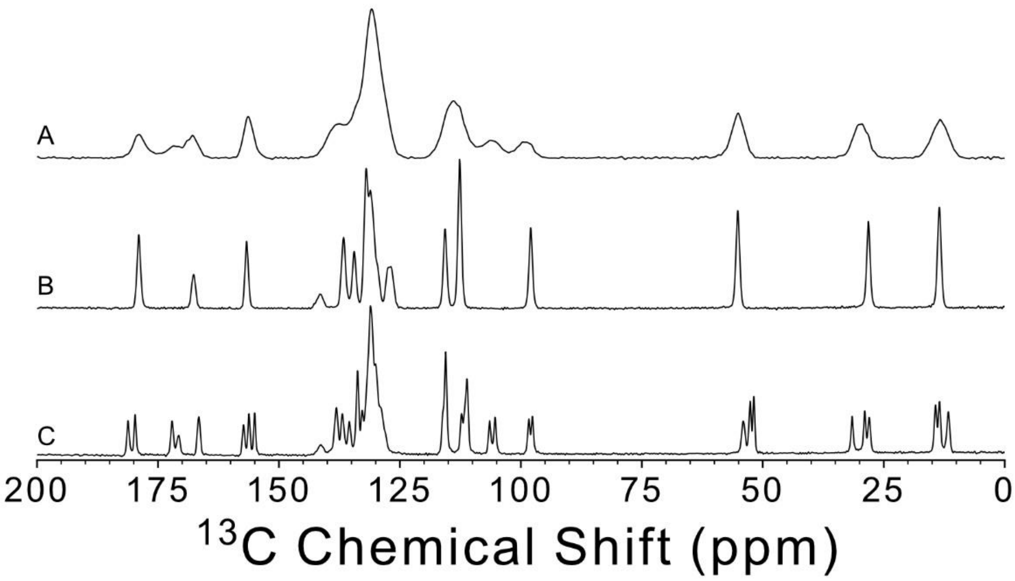

Figure S1. ^{1}H-^{13}C CP-MAS (n_r = 20 kHz) ssNMR spectra for (A) amorphous indomethacin, (B) gamma indomethacin, and (C) alpha indomethacin.

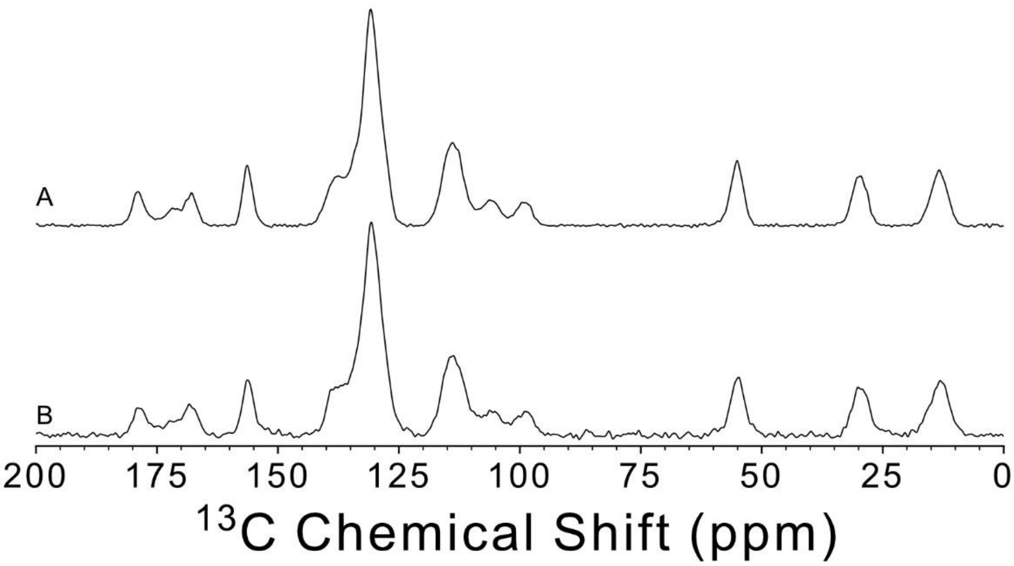

Figure S2. ^{1}H-^{13}C CP-MAS (n_r = 20 kHz) ssNMR spectra for amorphous (A) indomethacin, and (B) d_1-indomethacin.

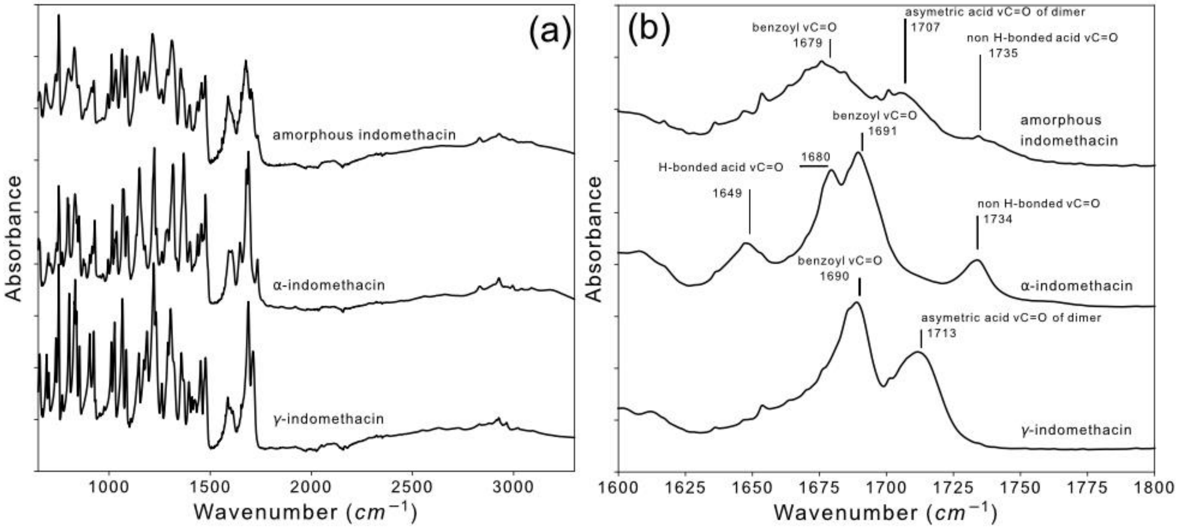

Figure S3. FT-IR spectra of Indomethacin polymorphs from 650 cm^{-1} to 3300 cm^{-1} (a) and the carbonyl stretching region from 1600 cm^{-1} to 1800 cm^{-1} (b). The carbonyl region contains assignments of characteristic stretching bands for each polymorph. These assignments are made in reference to the work of Van Duong, et al. [29]. ATR-FTIR spectra were collected from 650 cm^{-1} to 4000 cm^{-1} using an Agilent Cary 630 FTIR spectrometer with a diamond ATR. Samples were collected using 128 background and 128 sample scans with a resolution of 2 cm^{-1}. All samples were run as a powder at room temperature (293°C) and pressed in order to have consistent contact with the diamond ATR.

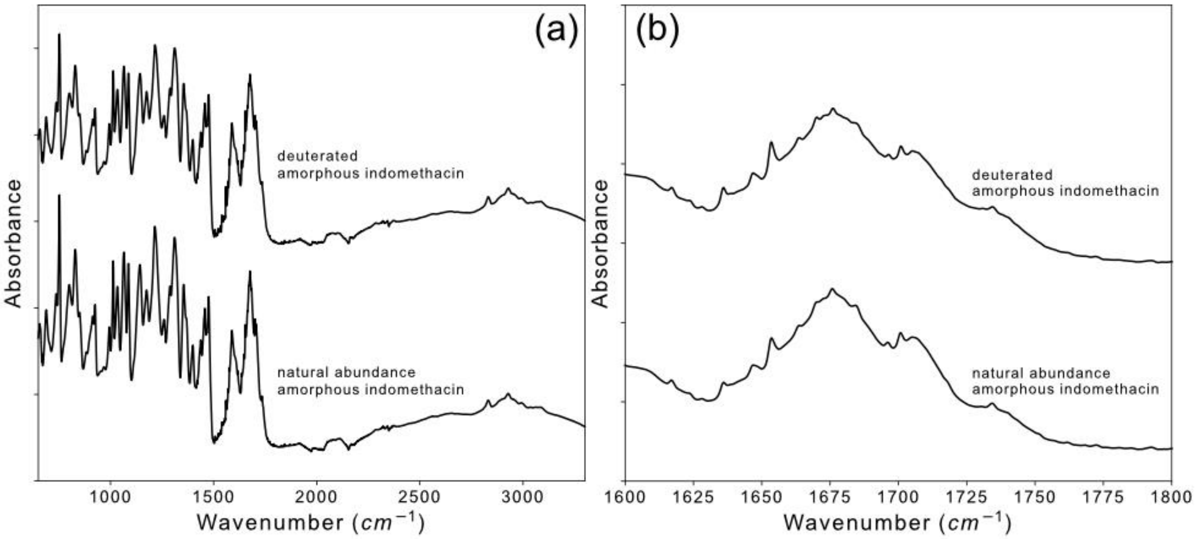

Figure S4. FT-IR spectra of deuterated vs. natural abundance gamma Indomethacin from 650 cm^{-1} to 3300 cm^{-1} (a) and in the carbonyl stretching region from 1600 cm^{-1} to 1800 cm^{-1} (b).

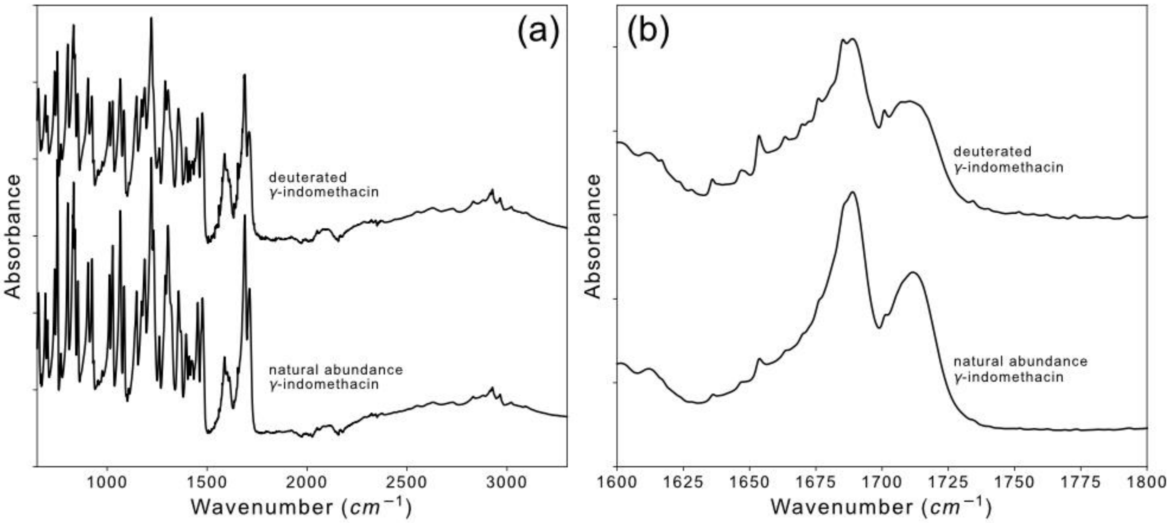

Figure S5. FT-IR spectra of deuterated vs. natural abundance amorphous Indomethacin from 650 cm^{-1} to 3300 cm^{-1} (a) and in the carbonyl stretching region from 1600 cm^{-1} to 1800 cm^{-1} (b).
